# A Population-Based Cohort Study on Peripheral Arterial Disease in Patients with Schizophrenia

**DOI:** 10.1371/journal.pone.0148759

**Published:** 2016-02-12

**Authors:** Wen-Yu Hsu, Cheng-Li Lin, Chia-Hung Kao

**Affiliations:** 1 Graduate Institute of Clinical Medical Science, China Medical University, Taichung, Taiwan; 2 Department of Psychiatry, Lutung Christian Hospital, LuKang, Taiwan; 3 Department of Psychiatry, School of Medicine, Chung Shan Medical University, Taichung, Taiwan; 4 Management Office for Health Data, China Medical University Hospital, Taichung, Taiwan; 5 College of Medicine, China Medical University, Taichung, Taiwan; 6 Graduate Institute of Clinical Medical Science and School of Medicine, College of Medicine, China Medical University, Taichung, Taiwan; 7 Department of Nuclear Medicine and PET Center, China Medical University Hospital, Taichung, Taiwan; University of Bologna, ITALY

## Abstract

**Purpose:**

Peripheral arterial disease (PAD) is considered the leading cause of atherosclerotic cardiovascular morbidity. Several risk factors of PAD have been observed in patients with schizophrenia. Therefore, we hypothesize that the incidence of PAD is higher in the schizophrenia population than in the general population.

**Methods:**

The patients in this population-based cohort study were selected from the Taiwanese National Health Insurance Research Database on the basis of the claims data from 2000 to 2011. We compared the incidence of PAD between schizophrenia and nonschizophrenia cohorts. Cox proportional hazard regression models were employed for analyzing the risk of PAD after adjustment for sex, age, and comorbidities.

**Results:**

The adjusted hazard ratio (HR) for PAD in the schizophrenia cohort was 1.26-fold higher than that in the nonschizophrenia cohort. Furthermore, patients with schizophrenia using atypical antipsychotics exhibited a high adjusted HR for PAD.

**Conclusion:**

Compared with the general population, the risk of PAD is higher among patients with schizophrenia. Early diagnosis and intervention can mitigate complications resulting from cardiovascular diseases and lower mortality.

## Introduction

Peripheral arterial disease (PAD) is the narrowing of arteries, mostly in the lower limbs, leading to claudication. The prevalence of PAD in both men and women has increased from 3% among those aged 25–29 years to 24% among those 95–99 years in high-income countries [1]. PAD is considered the leading cause of atherosclerotic cardiovascular morbidity, followed by coronary artery disease and stroke [2, 3]. The distal superficial femoral artery is the most common site of PAD occurrence, leading to claudication in the calf muscle region. However, patients do not always exhibit typical PAD symptoms. The incidence of asymptomatic PAD is higher than that of symptomatic PAD in the general population [4]. In a previous study, asymptomatic PAD was a crucial predictor of cardiovascular morbidity and mortality [5]. However, PAD is typically underrecognized and undertreated in clinical practice [6, 7]. The ankle—brachial index (ABI) is a simple noninvasive test that can identify adults at a high risk of PAD. Prevention and early management of PAD may lower the risk of myocardial infarction, stroke, or death. Several risk factors, such as old age, diabetes, smoking, dyslipidemia, obesity, and hypertension, are associated with PAD [6, 8]. Some modifiable risk factors may be helpful in the management of PAD [8], and some of these risk factors are easily identified in patients with schizophrenia.

Schizophrenia is a chronic, severe brain disorder causing disability. The global lifetime prevalence of schizophrenia is approximately 1%. Several studies have reported an increase in the risk of myocardial infarction, stroke, or death in the schizophrenia population [9–11]. Schizophrenia is associated with excess deaths from coronary heart disease and stroke in persons younger than 75 years. Cardiovascular disease occurs more frequently in people with schizophrenia and is the commonest cause of death [12]. Several PAD associated risk factors, such as smoking, low physical activity, diabetes, and hyperlipidemia, were higher prevalent in schizophrenia population. Several articles reported schizophrenia population has higher tobacco smoking rate than general population [13–15]. Smoking is the key risk factor related with PAD in our schizophrenia patients. Patients with schizophrenia have fewer physical activity [16]. Low physical activity might related with negative symptoms of schizophrenia. Patients with schizophrenia have higher prevalence of diabetes, and schizophrenia patients are at least double the risk of developing type 2 diabetes mellitus [15, 17]. And, history of diabetes can deeply influence PAD evolution and cardiovascular diseases [18]. Patients with schizophrenia also had a much higher prevalence of hyperlipidemia in young adulthood than that in the general population [19], and increased risk of initiation of anti-hyperlipidemia medications was noted among patients with schizophrenia [20]. Owing to sharing these risk factors, higher PAD in schizophrenia population was supposed. However, noepidemiological study has studied the relationship between PAD and schizophrenia.

The cause for higher PAD incidence in the schizophrenia population than in the general population remains unknown. It is crucial to understand this cause for the prevention and treatment of related diseases in the schizophrenia population. Early awareness of cardiovascular risk in this population can facilitate early intervention.

We hypothesize that the incidence of PAD is higher in the schizophrenia population than in the general population. Accordingly, we conducted this population-based cohort study in Taiwan to investigate the prevalence of PAD are between the schizophrenia and general populations.

## Methods

### Data Source

The Taiwan National Health Insurance (NHI) program was implemented in 1995 and offers comprehensive medical coverage to all residents of Taiwan (http://www.nhi.gov.tw/english/index.aspx). The Taiwanese National Health Research Institutes (NHRI) maintain the National Health Insurance Research Database (NHIRD), which contains the claims data of the enrollees. Before the electronic files are released for research purposes by the NHRI, the personal identification information is encrypted to protect patient privacy. The data used in this retrospective cohort study were obtained from the NHIRD. International Classification of Diseases, Ninth Revision, Clinical Modification (ICD-9-CM) codes were used for coding the diseases relevant to this study.

### Sampled Patients

All patients diagnosed with schizophrenia (ICD-9-CM code 295) from 2000 to 2011 were identified from the Registry of Catastrophic Illness Database (RCIPD) of the NHIRD, and the first schizophrenia diagnosis date was considered the index date. If a physician diagnosed and characterized schizophrenia in a patient as a catastrophic illness, then the patient can submit the related information and apply for a catastrophic illness certificate. Under the guidelines of the Ministry of Health and Welfare of Taiwan, the patient can submit this certificate to have copayments related to the illness waived for both outpatient and inpatient care. We excluded patients with a history of PAD (ICD-9-CM codes 440.0, 440.2, 440.3, 440.8, 440.9, 443, 444.0, 444.22, 444.8, 447.8, and 447.9) before the index date, and those with incomplete age or sex information. Patients in the nonschizophrenia cohort were randomly selected from the insured patients without any history of schizophrenia and PAD at baseline. The exclusion criteria for the patients in the nonschizophrenia cohort were identical to those for patients in the schizophrenia cohort. The nonschizophrenia cohort was frequency matched with the schizophrenia cohort on the basis of age (in 5-y bands), sex, the year of index date and comorbidities of diabetes, hypertension, hyperlipidemia, COPD, heart failure, CAD, stroke, obesity and asthma, and medication of statin and aspirin.

### Outcomes, Comorbidities, and Mediations

Both cohorts were followed until PAD diagnosis, censoring because of death, loss to follow-up, withdrawal from the insurance system, or the end of 2011. Preexisting comorbidities for each patient included diabetes (ICD-9-CM code 250), hypertension (ICD-9-CM codes 401–405), hyperlipidemia (ICD-9-CM code 272), chronic obstructive pulmonary disease (COPD) [ICD-9-CM codes 491, 492, 496]), heart failure (ICD-9-CM code 428), coronary artery disease (CAD [ICD-9-CM codes 410–414]), stroke (ICD-9-CM codes430-438), obesity (ICD-9-CM code 278) and asthma (ICD-9-CM code 493). Statin and aspirin were also analyzed between the schizophrenia patients and the nonschizophrenia cohort. In addition, we hypothesized that typical and atypical antipsychotics exert different effects on PAD in patients with schizophrenia.

### Ethics Statement

The NHIRD encrypts patient personal information to protect privacy and provides researchers with anonymous identification numbers associated with relevant claims information, including sex, date of birth, medical services received, and prescriptions. We excluded all individually identifying or patient demographic information. Therefore, the patient consent is not required to access the NHIRD. This study was approved by the Institutional Review Board (IRB) of China Medical University (CMUH104-REC2-115). The IRB specifically waived the consent requirement.

### Statistical Analysis

We compared the distributions of age, sex, comorbidities and medications between the schizophrenia and nonschizophrenia cohorts using the chi-square test. Student *t* test was used to compare the differences in the mean age and mean follow-up years between the 2 cohorts. The incidence density rate of PAD (per 10 000 person-y) was calculated on the basis of sex, age and, comorbidities for each cohort. Univariate and multivariate Cox proportional hazard regression models were employed to examine and compare the risk of PAD associated with schizophrenia between the 2 cohorts, and the risk was evaluated as a hazard ratio (HR) with a 95% confidence interval (CI). Multivariate models were employed after adjustment for age, sex, and comorbidities (diabetes, hypertension, hyperlipidemia, COPD, heart failure, CAD, stroke, obesity and asthma) and medication of statin and aspirin. Further analysis was performed to assess the role of antipsychotics in influencing the PAD outcomes. The Kaplan—Meier method was employed for estimating the cumulative incidence of PAD between the schizophrenia and nonschizophrenia cohorts, and the differences were assessed using a log-rank test. Statistical analysis was performed using the SAS 9.3 statistical package (SAS Institute Inc., NC, USA), and *P*<0.05 in 2-tailed tests was considered significant.

## Results

**Fig 1:**
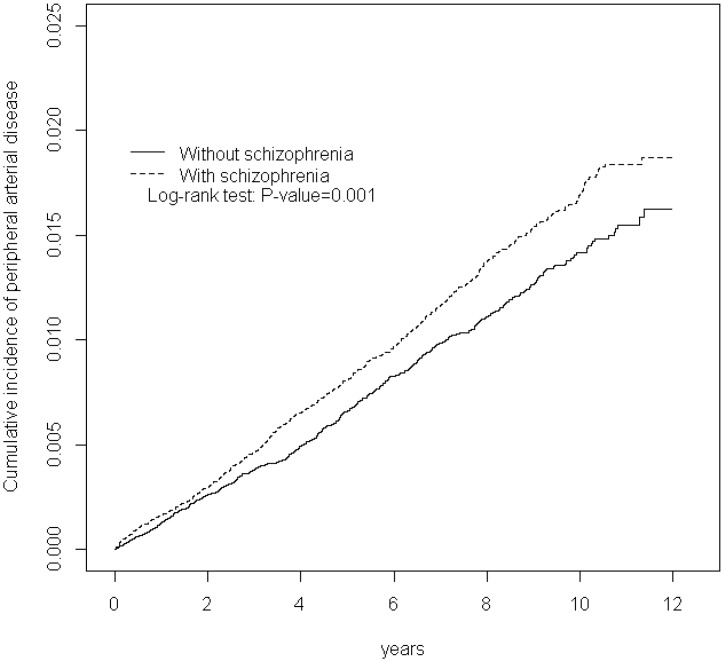
Cummulative incidence of peripheral arterial occlusive disease in patients with schizophrenia and comparison patients.

We identified 59 234 patients with newly diagnosed schizophrenia from 2000 to 2011 as the schizophrenia cohort and 59 234patients without any schizophrenia diagnosis as the nonschizophrenia cohort (Table 1). The mean (± standard deviation [SD]) age of patients in the schizophrenia and nonschizophrenia cohorts was 38.6(±13.2) and 38.5(±13.6) years, respectively, and approximately 45% of these patients were in the age group of 20–34 years. The number of men (52.3%) was higher than that of women (47.3%). The prevalence of preexisting comorbidities (diabetes, COPD, heart failure, stroke, obesity and asthma)and statin use and aspirin use were similar in the schizophrenia cohort than in the nonschizophrenia cohort. The mean follow-up duration was 6.40 (SD = 3.37) and 6.46 (SD = 3.32) years for the schizophrenia and nonschizophrenia cohorts, respectively. Fig 1 shows that the cumulative incidence of PAD was higher in the schizophrenia cohort than in the nonschizophrenia cohort (log-rank test, *P* = 0.001). The overall incidence of PAD was 1.26-fold higher in the schizophrenia cohort than that in the nonschizophrenia cohort (16.8 and 13.9 per 10 000 person-years, respectively), with a crude HR of 1.21 (95% CI = 1.08–1.36, Table 2). After adjustment for age, sex, comorbidities (diabetes, hypertension, hyperlipidemia, COPD, heart failure, CAD, stroke, obesity and asthma) and medication of statin and aspirin, patients in the schizophrenia cohort had a 1.26-fold higher risk of PAD than those in the nonschizophrenia cohort (HR = 1.26, 95% CI = 1.13–1.42). Sex-specific analysis showed that the risks of PAD were higher in women than in men, and these risks were higher in the schizophrenia cohort than in the nonschizophrenia cohort (HR = 1.08, 95% CI = 1.03–1.14 for men; HR = 1.50, 95% CI = 1.26–1.77, *P* for interaction = 0.01). The incidence densities of PAD increased with age in both cohorts. The age-specific schizophrenia-to-nonschizophrenia relative risk of PAD was higher for younger patients (age = 20–34 y, HR = 1.72, 95% CI = 1.25–2.37) than for older patients (age >50 y, HR = 1.06, 95% CI = 0.89–1.26, *P* for interaction = 0.02). The comorbidity-specific schizophrenia-to-nonschizophrenia risk of PAD was significantly higher for patients without comorbidities (HR = 1.39, 95% CI = 1.18–1.65). The incidence densities of PAD increased for patients with statin use or aspirin use in both cohorts. The risk of PAD in schizophrenia patients had nearly 1.25-fold higher than the risk in nonschizophrenia cohort by follow-up ≤31 year (HR = 1.25, 95% CI = 1.20–1.30). The PAD risk in schizophrenia patient were 1.22, and 1.40-fold by 4–6 years (HR = 1.22, 95% CI = 1.17–1.28), and 7–9 years (HR = 1.40, 95% CI = 1.31–1.49), respectively.

**Table 1:** Comparison of demographics and comorbidity between Schizophrenia patients and controls.

	Control subjects (N = 59234)	Schizophrenia (N = 59234)	
	n(%)	n(%)	*p*-value
**Age, years**			0.45
20–34	26500(44.7)	26625(45.0)	
35–49	21308(36.0)	21352(36.1)	
>50	11426(19.3)	11257(19.0)	
Mean (SD) ^†^	38.5(13.6)	38.6(13.2)	0.51
**Gender**			0.83
Female	28055(47.4)	28017(47.3)	
Male	31179(52.6)	31217(52.7)	
**Comorbidity**			
Diabetes	2343(3.96)	2375(4.01)	0.63
Hypertension	6622(11.2)	6597(11.1)	0.82
Hyperlipidemia	4053(6.84)	4042(6.82)	0.90
COPD	5056(8.54)	4944(8.35)	0.24
Heart failure	323(0.55)	353(0.60)	0.25
CAD	2453(4.14)	2483(4.19)	0.66
Stroke	788(1.33)	796(1.34)	0.84
Obesity	679(1.15)	677(1.14)	0.96
Asthma	2436(4.11)	2380(4.02)	0.41
**Medication**			
Statin	1641(2.77)	1640(2.77)	0.99
Aspirin	7809(13.2)	7814(13.2)	0.97

Chi-square test examined categorical data;

^†^T-test examined continuous.

**Table 2:** Incidence and adjusted hazard ratio of peripheral arterial disease by sex, age and comorbidity for Schizophrenia patients compared to controls.

	Control subjects			Schizophrenia			Compared to Control	
Variables	Events	PY	Rate^#^	Events	PY	Rate^#^	Crude HR*(95% CI)	Adjusted HR^†^(95% CI)
**All**	531	382747	13.9	635	378809	16.8	1.21(1.08, 1.36)**	1.26(1.13, 1.42)***
**Gender**								
Female	299	178759	16.7	311	176805	17.6	1.05(1.00, 1.11)	1.08(1.03, 1.14)**
Male	232	203988	11.4	324	202004	16.0	1.41(1.19, 1.67)***	1.50(1.26, 1.77)***
P for interaction								0.01
**Age, years**								
20–34	59	174813	3.38	109	177462	6.14	1.82(1.32, 2.49)***	1.72(1.25, 2.37)***
35–49	210	139686	15.0	272	137799	19.7	1.32(1.10, 1.58)**	1.31(1.09, 1.57)**
>50	262	68247	38.4	254	63547	40.0	1.04(0.88, 1.24)	1.06(0.89, 1.26)
P for interaction								0.02
**Comorbidity**^§^								
No	228	299448	7.61	309	298630	10.4	1.36(1.15, 1.61)***	1.39(1.18, 1.65)***
Yes	303	83299	36.4	326	80178	40.7	1.12(0.96, 1.31)	1.16(0.99, 1.36)
P for interaction								0.10
**Medication**								
Statin								
No	492	375371	13.1	597	371575	16.1	1.23(1.09, 1.38)***	1.28(1.13, 1.44)***
Yes	39	7375	52.9	38	7234	52.5	0.99(0.63, 1.55)	1.04(0.66, 1.62)
P for interaction								0.38
**Aspirin**								
No	406	338565	12.0	484	335962	14.4	1.20(1.05, 1.37)**	1.24(1.09, 1.42)**
Yes	125	44182	28.3	151	42846	35.2	1.25(0.98, 1.58)	1.32(1.04, 1.68)*
P for interaction								0.79
**Follow-up time**								
≤3	204	160300	12.7	245	159559	15.4	1.21(1.16, 1.26)***	1.25(1.20, 1.30)***
4–6	181	122746	14.8	208	120314	17.3	1.17(1.12, 1.23)***	1.22(1.17, 1.28)***
7–9	113	28061	40.3	148	27172	54.5	1.35(1.26, 1.45)***	1.40(1.31, 1.49)***
>9	33	22413	14.7	34	23166	14.7	1.00(0.92, 1.08)	1.05(0.97, 1.14)

PY, person-years;

^#^, incidence rate, per 10,000 person-years;

*: relative hazard ratio;

^†^: adjusted hazard ratio controlling for age, gender, and comorbidities of diabetes, hypertension, hyperlipidemia, COPD, heart failure, CAD, stroke, obesity and asthma, and medication of statin and aspirin;

^§^: Patients with any one of the comorbidities diabetes, hypertension, hyperlipidemia, COPD, heart failure, CAD, stroke, obesity and asthma were classified as the comorbidity group;

*p<0.05,

**p<0.01,

***p<0.001.

Table 3 shows the association of the risk of PAD with treatments between the schizophrenia and nonschizophrenia cohorts. The patients in the schizophrenia cohort were at a significantly higher risk of PAD than those in the nonschizophrenia cohort. Among the patients in the schizophrenia cohort, those who received combined typical and atypical antipsychotic treatment exhibited a significantly higher risk of PAD (HR = 1.86, 95% CI = 1.34–2.59) than those in the comparison cohort, followed by those who received only atypical antipsychotic treatment (HR = 1.28, 95% CI = 1.14–1.44) than those in the nonschizophrenia cohort. Compared with the nonschizophrenia cohort, schizophrenia who received atypical antipsychotics treatment for less than 1 year were associated with higher risk of PAD (HR = 1.55, 95% CI = 1.30–1.86), followed by those who received atypical antipsychotic treatment more than 1 year (HR = 1.19, 95% CI = 1.05–1.36).

**Table 3:** Incidence and hazard ratio of peripheral arterial occlusive disease for schizophrenia patients with treatment compared with non-schizophrenia subjects.

Variables(ICD-9 code)	Event	Rate^#^	Crude HR*(95% CI)	Adjusted HR^†^ (95% CI)
Non-schizophrenia	531	13.9	1(Reference)	1(Reference)
Atypical antipsychotics	537	17.2	1.24(1.10, 1.40)***	1.28(1.14, 1.44)***
Typical antipsychotics	60	11.5	0.84(0.64, 1.10)	0.96(0.73, 1.25)
Both	38	25.7	1.84(1.32, 2.56)***	1.86(1.34, 2.59)***
Schizophreniawith Atypical antipsychotics ≤ 1 y	151	21.1	1.53(1.28, 1.84)***	1.55(1.30, 1.86)***
Schizophrenia with Atypical antipsychotics> 1 y	386	16.1	1.16(1.01, 1.32)*	1.19(1.05, 1.36)**
Schizophreniawith Typical antipsychotics ≤ 1 y	16	16.6	1.22(0.74, 2.01)	1.18(0.72, 1.95)
Schizophrenia with Typical antipsychotics> 1 y	44	10.3	0.76(0.56, 1.04)	0.90(0.66, 1.22)

PY, person-years;

^#^, incidence rate, per 10,000 person-years;

*: relative hazard ratio;

^†^: adjusted hazard ratio controlling for age, sex, and comorbidities of diabetes, hypertension, hyperlipidemia, COPD, heart failure, CAD, stroke, obesity and asthma, and medication of statin and aspirin;

*p<0.05,

**p<0.01,

***p<0.001.

## Discussion

Our results show that the patients in the schizophrenia cohort have a 1.26-fold higher risk of PAD than those in the nonschizophrenia cohort after controlling for age, gender, and comorbidities of diabetes, hypertension, hyperlipidemia, COPD, heart failure, CAD, stroke, obesity and asthma, and medication of statin and aspirin. Peripheral vasculature has not been explored exclusively in patients with schizophrenia. This is the first study to report that patients with schizophrenia have a higher risk of PAD than their comparable controls. Patients with PAD are at a high risk of adverse cardiovascular events [21]. PAD, a manifestation of systemic atherosclerosis, leads to segmental narrowing and occlusion of arteries. Our results are in agreement with those of Israel et al, in which peripheral endothelial dysfunction was reported in unmedicated patients with acute schizophrenia [22]. Besides, schizophrenia patients have higher prevalence of tobacco smoking, low physical activity, diabetes, and hyperlipidemia. These would worst their vascular health.

Our results showed that the incidence rate of PAD was higher in women than in men in the nonschizophrenia cohort, which is similar to those of previous reports [23, 24]. In addition, we found that the risk of PAD increased in both men and women in the schizophrenia cohort. Men in the schizophrenia cohort had a 1.50-fold higher adjusted HR for PAD than those in the nonschizophrenia cohort. Women in the schizophrenia cohort had a 1.08-fold higher adjusted HR for PAD than those in the nonschizophrenia cohort. In a previous study, the rate of smoking was higher in men than in women in patients with schizophrenia [25]. Early onset of schizophrenia in males might result in early process of peripheral endothelial dysfunction. A high rate of smoking and early onset age of schizophrenia in men in the schizophrenia cohort may explain our results and the rate of smoking was accounted for by adjusting for smoking-associated diseases (such as asthma).

Age plays a crucial role in PAD. The prevalence of PAD increased in older patients in a previous study [1]. In our study, we observed that the incidence of PAD was higher in older patients in both the cohorts. However, patients aged between 20 and 34 years in the schizophrenia cohort have a 1.72-fold higher adjusted HR for PAD than those in the nonschizophrenia cohort. Age between 35 and 49 schizophrenia cohort have a 1.31-fold higher adjusted HR for PAD development than the age between 35 and 49 years in the nonschizophrenia cohort. No significant difference in both the age more than 50 year old cohorts. These results indicate that younger patients with schizophrenia have a high PAD risk. The results of our study showed that schizophrenia may facilitate the progress of atherosclerosis and is consistent with the report of Israel et al [22], in which peripheral endothelial dysfunction was reported in unmedicated patients with schizophrenia. The anti-inflammatory mediator nitric oxide exerts a protective effect on endothelial functioning. Burghardt et al suggested that patients with schizophrenia may lose the genetic protection provided by nitric oxide once their condition progresses to the pro-inflammatory state of metabolic syndrome [26].

In our study, we investigated the effect of different antipsychotics. We found that patients in the schizophrenia cohort who received atypical antipsychotic treatment had a higher HR for PAD (1.28) than those in the nonschizophrenia cohort after adjustment for age, sex, and comorbidities of diabetes, hypertension, hyperlipidemia, COPD, heart failure, CAD, stroke, obesity and asthma, and medication of statin and aspirin. No significant difference was observed for typical antipsychotic treatment after adjustment for confounding factors between the schizophrenia and nonschizophrenia cohorts for PAD. Compared with patients in the nonschizophrenia cohort, patients in the schizophrenia cohort who received combined typical and atypical antipsychotic treatment had a higher HR (1.86) for PAD after adjustment. Antipsychotics, particularly atypical antipsychotics, may play a role in the development of PAD. The Clinical Antipsychotic Trials of Intervention Effectiveness study revealed an association between the use of atypical antipsychotics in schizophrenia and metabolic syndrome [27]. The American Diabetes Association, the American Psychiatric Association, the American Association of Clinical Endocrinologists, and the North American Association for the Study of Obesity suggest that baseline screening and follow-up monitoring is essential for mitigating the likelihood of developing CVD, diabetes, or other diabetes-related complications while prescribing atypical antipsychotics [28]. Atypical antipsychotics may counteract some vascular health benefits of a diet high in omega 3 fatty acids, as reported in the study by Ellingrod [29]. Our study results were in agreement with those of the aforementioned reports.

However, some limitations were encountered while conducting this study. First, the accuracy of the incidence of PAD was not validated by reviewing the medical charts, and PAD cases were identified only on the basis of the ICD-9-CM codes(440.0, 440.2, 440.3, 440.8, 440.9, 443, 444.0, 444.22, 444.8, 447.8, and 447.9). Second, the use of ICD system should be considered as a limitation of this study. The use of ICD could be considered as a further limitation of this study. However, the NHIRD covers a highly representative sample of Taiwan’s general population because the reimbursement policy is universal and operated by a single-buyer, the government in Taiwan. All insurance claims should be scrutinized by medical reimbursement specialists and peer review according to the standard diagnosed criteria in the study. If these doctors or hospitals make wrong coding for the diagnoses, they will be punished with a lot of penalties. Therefore, the diagnoses based on ICD-9 codes in this study should be highly reliable. Third, the incidence of asymptomatic PAD is higher than that of symptomatic PAD in the general population. In our study, asymptomatic PAD was not considered as part of the study design. Fourth, lack of data (e.g, smoking status, obesity, and family history) may have influenced the results of our study. Therefore, we applied proxy measures, such as hyperlipidemia and diabetes as indicators of obesity and COPD as an indicator of smoking, for controlling the potential confounding effects. However, the effects of some unmeasured confounders could not be addressed in our study. Fifth, the adherence of treatment (statin and aspirin) in schizophrenia cohort might be poorer than in nonschizophrenia cohort. Sixth, the experimental design is another limitation. Our study is a retrospective cohort study. The evidence derived from a retrospective cohort study is generally of lower methodological quality than that from randomized trials by the experimental design because a retrospective cohort study is subject to many biases related to the necessary adjustments for confounding factors. Despite the meticulous design of this study and its adequate control of confounding factors, biases could remain because of possibly unmeasured or unknown confounding factors.

In conclusion, this nationwide population-based cohort study shows that patients in the schizophrenia cohort have a 1.26-fold higher adjusted HR for PAD than that in the nonschizophrenia cohort after adjustment. This is the first study to report the association between schizophrenia and the risk of PAD. Patients with schizophrenia who were treated using atypical antipsychotics have a high risk of PAD. However, most patients with the new onset schizophrenia patients received atypical antipsychotics. It is crucial to adequately assess the risk of PAD among patients with schizophrenia, particularly those who are unable to exercise adequate personal care or gain access to health care by themselves because of their illness. In-depth ABI analyses may facilitate PAD diagnosis. Early diagnosis and management of PAD may further facilitate the prevention of myocardial infarction, stroke, and other cardiovascular diseases. Additional studies are warranted to examine the role of PAD in schizophrenia.

## Abbreviations

aHR: adjusted hazard ratio
CI: confidence interval
ICD-9-CM: International Classification of Diseases, Ninth Revision, Clinical Modification
NHIRD: National Health Insurance Research Database
LHID 2000: Longitudinal Health Insurance Database 2000
PAD: peripheral arterial disease
